# Exome/Genome-Wide Testing in Newborn Screening: A Proportionate Path Forward

**DOI:** 10.3389/fgene.2022.865400

**Published:** 2022-07-04

**Authors:** Vasiliki Rahimzadeh, Jan M. Friedman, Guido de Wert, Bartha M. Knoppers

**Affiliations:** ^1^ Stanford Center for Biomedical Ethics, Stanford University, Stanford, CA, United States; ^2^ Department of Medical Genetics, University of British Columbia, Vancouver, BC, Canada; ^3^ Department of Health, Ethics and Society, Maastricht University, Maastricht, Netherlands; ^4^ Centre of Genomics and Policy, McGill University, Montreal, QC, Canada

**Keywords:** exome sequencing, genome sequecing, newborn screening, population health genomics, access, public health ethics

## Abstract

Population-based newborn screening (NBS) is among the most effective public health programs ever launched, improving health outcomes for newborns who screen positive worldwide through early detection and clinical intervention for genetic disorders discovered in the earliest hours of life. Key to the success of newborn screening programs has been near universal accessibility and participation. Interest has been building to expand newborn screening programs to also include many rare genetic diseases that can now be identified by exome or genome sequencing (ES/GS). Significant declines in sequencing costs as well as improvements to sequencing technologies have enabled researchers to elucidate novel gene-disease associations that motivate possible expansion of newborn screening programs. In this paper we consider recommendations from professional genetic societies in Europe and North America in light of scientific advances in ES/GS and our current understanding of the limitations of ES/GS approaches in the NBS context. We invoke the principle of proportionality—that benefits clearly outweigh associated risks—and the human right to benefit from science to argue that rigorous evidence is still needed for ES/GS that demonstrates clinical utility, accurate genomic variant interpretation, cost effectiveness and universal accessibility of testing and necessary follow-up care and treatment. Confirmatory or second-tier testing using ES/GS may be appropriate as an adjunct to conventional newborn screening in some circumstances. Such cases could serve as important testbeds from which to gather data on relevant programmatic barriers and facilitators to wider ES/GS implementation.

## Introduction

Population-based newborn screening (NBS) is among the most effective public health programs ever launched (Tonniges, 2000; Sahai and Marsden, 2009; Berry, 2015). Updated national estimates in the United States suggest nearly 12,900 newborns screened positive for childhood onset disorders that previously led to severe morbidity or mortality and were listed on the Recommended Universal Screening Panel (RUSP) (5) between 2015 and 2017 (Sontag et al., 2020). Key to the success of NBS programs has been their affordability and near universal access and participation. Pre-symptomatic treatment of newborns who screen positive for some of these conditions is much more cost-effective and less burdensome on healthcare systems than treating the conditions once they become symptomatic (Carroll and Downs, 2006). Preventing the development of symptomatic disease is a particularly important consideration with respect to genetic diseases that can be detected by ES/GS analysis because most do not have specific treatments that can prevent disease onset or progression.

Since early validation studies of mass screening tests for metabolic disorders in the 1960s (McCandless and Wright, 2020), NBS methods as well as their formal adoption and oversight have evolved considerably. Interest has been building to expand NBS programs to also include more rare genetic diseases that can be identified using ES/GS approaches (Holm et al., 2018; Genomics England and the UK National Screening Committee, 2021; Gold et al., 2022; Lu et al., 2022). Improvements to genome sequencing technologies that enable researchers to elucidate novel gene-disease associations and to diagnose conditions undiscoverable using traditional biochemical or other biomarker testing, and the wide availability and declining costs of genomic testing are among the reasons ES/GS might be advantageous as a first-tier clinical test for diagnosing genetic diseases.

At the outset, it is important to distinguish NBS meant to identify pre-symptomatic infants rare but potentially devastating conditions e.g., phenylketonuria (PKU), severe combined immunodeficiency disease of congenital heart defects, from screening for risk stratification meant to guide lifestyle modification or surveillance protocols routinely offered to adults. Current universal NBS protocols fall into the first category; ES/GS of newborn infants for most genetic diseases would fall into the second category. This is true whether one considers all known genetic diseases or only a subset in which non-specific interventions may be able to reduce the risk or age of symptomatic onset.

Using ES/GS as a tool in NBS may also inappropriately conflate the recognition of a disease-associated genetic variant with diagnosis of the disease. Diagnosing a genetic disease requires a physician to interpret an ES/GS result in the context of an individual’s complete clinical picture–the medical history, family history, physical exam, and other laboratory and imaging studies–in light of what is known about the range of clinical manifestations, inheritance pattern, penetrance, and variability of the disease. Complete clinical assessment is the only confirmatory “test” available for most genetic diseases. If universal NBS relied on sequencing the entire genome, exome or specific regions of the exome, then complete clinical assessment for the genetic disease indicated would be necessary to confirm the molecular “diagnosis” in every case. Population-based NBS of any kind should only be offered as part of a comprehensive public health program that includes clinical follow-up, therapeutic interventions, quality assurance, governance and oversight, and public and professional education (Friedman et al., 2017) in addition to the confirmatory complete clinical assessment and genetic counselling (if the condition found is a genetic disease). If ES/GS is being considered as a replacement for current NBS, evidence that the ES/GS methods are superior to the existing methods is necessary. Adoption of sequencing-based NBS without consideration of the unique ethical, legal and social issues it raises (Eichinger et al., 2021; Woerner et al., 2021) risks widening disparities in availability and access to standard NBS, particularly in under-resourced settings.

In this paper, we review recommendations from professional bodies regarding integration of genomic sequencing methods in public NBS programs in Europe (Howard et al., 2015) and North America, where the authors are based. We limit our discussion of relevant ethical, legal and social issues associated with universal ES/GS as a population screening tool for newborns, acknowledging, as others do (Johnston et al., 2018), that different professional obligations and standards exist in clinical screening, diagnostic, and direct-to-consumer contexts. Our analysis focuses on applications of universal genomic sequencing of the genome, exome, or a portion of the exome that includes a large number of disease-associated genes. We refer to as “ES/GS,” rather than on targeted sequencing of one or a few genes for confirmatory testing of conditions identified by conventional NBS (Bhattacharjee et al., 2015).

Indeed, there are compelling advantages for supporting genomic sequencing method applied in the NBS context. Genomic sequencing has been shown to detect previously fatal diseases in affected newborns, as well as provide information to patients and families about genetic predisposition risks for later onset diseases (Holm et al., 2018) and inform preventative clinical action. Scholars have also argued that biological family may receive ancillary benefits from recognition of disease-associated variants in an infant by enabling prenatal diagnosis or specialized care for future pregnancies, earlier diagnosis or prevention of disease in relatives, or the empowerment provided by better knowledge (Ceyhan-Birsoy et al., 2019; Biesecker et al., 2021). However, the “gap between what sequencing results can reveal and the kinds of information most people need to improve their health, combined with widely publicized hopes for the revolutionary power of genomics, creates the very real risk that patients, research participants, health care professionals, policy-makers, and others may have unrealistic expectations of what sequencing can achieve and little appreciation for its downsides” (Johnston et al., 2018).

Public opinion research suggests that family preferences vary considerably regarding whether and how to return genomic sequencing results (Lipstein et al., 2010; Fernandez et al., 2014; Botkin et al., 2015; Joseph et al., 2016; Pereira et al., 2021), to say nothing of current shortages of genetic counsellors and genetic specialist physicians needed or enhancements to genomic literacy and education for health professionals and the general public should ES/GS become routine in NBS (Lewis et al., 2016). Key policy questions also remain unresolved. These include: What rights and protections apply for genomic and related health data involving newborns when they become adults? How will public health agencies ensure that appropriate infrastructures for sequencing, variant interpretation, diagnostic confirmation, treatment or non-medical interventions, genetic counselling, clinical follow-up, and program governance and quality assurance are in place and accessible to all infants, even those in under-resourced settings? And whether requirements for explicit informed consent to ES/GS-based NBS would need to obtained from the parents and, if so, should it include permission for others (researchers, family members, police, etc.) to access stored newborn sequencing data in the future.

We assess these questions by evaluating the proposed benefits and foreseeable risks of implementing ES/GS in NBS. In our analysis, we apply the principle of proportionality to our discussion—that benefits of sequencing should clearly outweigh associated risks—and consider the human right to benefit from science -especially that of the asymptomatic, at-risk newborn to be found. We conclude that routine universal ES/GS implementation is not justified at the present time, even if the analysis is restricted to a subset of disease-associated genes. Stronger evidence is needed to establish the clinical utility of ES/GS, accurate genomic variant interpretation, and cost effectiveness for newborn screening, as well as policies ensuring universal access and equitable resourcing for not only the testing but also for comprehensive diagnostic confirmation, treatment, genetic counselling, and clinical follow-up of affected patients. Moreover, this evidence should demonstrate the population health benefits of universal ES/GS-wide screening of newborns and not simply that anticipated harms of incorporating ES/GS are minimal. Prioritizing expanded access over expanded testing is likely to lead to more equitable distribution of the public health benefits of newborn screening programs.

## Principle of Proportionality

The principle of proportionality suggests an intervention may be ethically permissible if its anticipated benefits on balance justify exposure to associated harms and hence a helpful framework with which to assess ES/GS-based screening (Sénécal et al., 2018). The principle is rooted in moral and legal theory of punishment. 17th Century constitutional law theorists, for example, invoked the principle to judge the statutory fairness between restrictions imposed to implement a corrective measure and the severity of the act(s) the measure purports to mitigate (Walen, 2021). In research, the proportionality principle underpins decisions institutional/ethics review boards make regarding the relative risks and benefits of a study to prospective participants and is subsequently codified in national human subjects research regulations (OHRP, 2017; Canadian Institutes of Health Research, 2018) and international biomedical research norms (Council for International Organizations of Medical Sciences (CIOMS) in collaboration with the World Health Organization, 2016; WMA, 2022). It has also been has more recently been applied to guide privacy protections when sharing genomic and related health data (Wright et al., 2016).

And last, but not least, some more recent versions of the normative framework for screening add the principle of proportionality as a central, over-arching, screening criterion: “The overall benefits of screening should outweigh the harm” (Andermann et al., 2008; Health Council of the Netherlands, 2008). The appeal of the proportionality principle to the NBS debate is astutely summarized by Kalkman and Dondorp in their position against screening newborns for non-treatable conditions: “the dividing line in the debate is … whether such screening should be regarded as catering to a parental “right to know,” or as a public health service that should be subject to standards of evidence and proportionality” (Kalkman and Dondorp, 2022).

### The Benefits of Accurate and Timely Diagnosis

New precision methods to detect disease-causing genetic variants have greatly improved (Dondorp and de Wert, 2013). ES/GS could identify infants with rare genetic diseases not currently recognized using standard NBS. In theory, newborns who screen positive by ES/GS have the potential to benefit from: early diagnosis; disease onset prevention using available approaches; opportunities for genetic counselling for their families; eligibility for participation in clinical trials or other research studies; and avoiding long and difficult diagnostic odysseys.

ES/GS should not, in our view, replace standard methods for any disease screening unless the former has been shown to have better sensitivity and specificity for the disease. For conditions that are not included in current NBS programs, development and uniform adoption of an approach will be needed to select the conditions for which ES/GS are expected to provide tangible benefit to the newborn. An exome- or genome-wide analysis that generates more harms than benefits or for which the harms and benefits have not been established is ethically unjustifiable–a more targeted analysis is to be preferred; see for example (Milko et al., 2019). But agreement on a uniform approach for selecting conditions detectable only using ES/GS is proving elusive for NBS programs worldwide (Jansen et al., 2017). Assuming agreement on the approach were achieved, the question would become whether every disease gene that we look for using ES/GS must meet the same criteria required to add conditions to the RUSP.

The benefit-harm calculus is further complicated by the type of disorder being screened. One significant challenge facing public health decision-makers and clinicians alike is determining when to add conditions to the RUSP that are identifiable only through ES/GS methods. For diseases for which standard screening is superior, ES/GS may be considered as an add-on to current first-tier screening programs. Findings from a comparison study for example showed that traditional NBS using tandem mass spectrometry had greater sensitivity and specificity than ES for the diseases that are currently being screened, but ES was useful for confirmatory (Adhikari et al., 2020).

### Screening for Late-Onset Conditions

Debates abound in the literature regarding the ethics of testing children for conditions likely to present later in life or which may be clinically relevant for parents or other biological family members in the immediate term. The presumption of clinical benefit to the parents and family members, however, has been challenged (Buchbinder and Timmermans, 2011; Ross and Clayton, 2019). Screening parents themselves using ES/GS for previously unrecognized conditions would not only be more clinically effective but, most importantly, avoids instrumentalizing the child for parental benefit. We furthermore object to predictive testing for later-onset disorders taking account both the harm principle and the principle of respect for the child’s future right to informational self-determination, a specification of the child’s proposed right to an open future (Davis, 1997). Professional guidelines are consistent with these principles, advocating that publicly funded, universal NBS should be limited to diseases that can be diagnosed in the newborn period and which can be effectively treated or prevented during childhood (de Wert et al., 2021; Miller et al., 2021). As others have argued, “Providing additional genomic information beyond the most actionable conditions, while potentially of interest to many parents, may increase the complexity of informed consent and thereby serve to distract from the primary health benefits” (Roman et al., 2020). Broadening the scope of NBS beyond its primary aim of detecting rare disorders in asymptomatic children has the potential to adversely impact the universal delivery of NBS, to say nothing of the impacts on public trust and widespread support for NBS.

### Testing Capability and Challenges in Genomic Variant Interpretation

Standard clinical analyses of ES/GS data do not reliably identify some kinds of disease-causing genetic variants, including short tandem repeat expansions, mobile element insertions, and complex or small structural variants. Knowing that ES/GS-based NBS has been done may preclude or delay appropriate genetic testing for symptomatic genetic disease in an older child or adult.

Interpretation of NBS results requires extensive knowledge of benign, as well as disease-causing variants for every gene tested. The sensitivity and specificity of ES/GS for most rare genetic diseases are unknown and likely to remain so because sample sizes are small and studies difficult to power sufficiently. In addition, the penetrance and phenotypic spectrum associated with pathogenic variants for most genetic disease loci are unknown. Thus, it is difficult or impossible to know if an asymptomatic baby with a “molecular diagnosis” of a rare genetic disease will ever develop the disease or, in the event the child does develop the disease, when it will occur or how severe it will be. Moreover, genetic disease diagnosis is Bayesian. That is, the probability of finding a pathogenic variant is small in a healthy newborn with no family history of the genetic disease. Since there is no primary indication for NBS, the *a priori* risk that an infant will develop any particular genetic disease is extremely small. This makes “positive” results more likely to be false positives and less likely to be true positives, even if the analytical validity of the test is very high.

Our inability at the present time to interpret the pathogenicity of most genomic variants is perhaps the strongest reason against adopting ES/GS in population-based NBS, despite improvements to clinical annotation of variants (Amendola et al., 2020) and broader accessibility to relevant databases at the point of care (Rehm et al., 2015). The problems of interpretation also exacerbate the effects of false positives/negatives on families and the healthcare system that are likely to result if variants of hundreds or thousands of potential disease genes are analyzed (Adhikari et al., 2020).

The confidence of variant classification and clinical interpretation of genetic results will determine their predictive value. In line with the ethical principle of proportionality, proponents of ES/GS-based NBS will need to specify thresholds for what genes and/or variants should be disclosed in a screening context based on better understanding of anticipated benefits and harms associated with those decisions. The general issue remains that ES/GS is currently used as a diagnostic test, i.e., to confirm a clinical diagnosis of suspected genetic disease. However, in NBS, ES/GS would be used as a screening test to identify children who are at high risk of a genetic disease implied by the “molecular diagnosis.” If ES/GS were indeed used as a screening test, confirmatory testing to manage the inevitable false positives must be available. The distinction between the ES/GS result, regardless of its ACMG classification, and the actual diagnosis of a disease in the child would have to be explicit, generally accepted, and universally understood to avoid stigmatization, discrimination, insurance coverage, among other social issues.

Interpretation of ES/GS variants requires comparisons to allele frequencies in both diagnosed and healthy populations and has direct implications for justice and health equity. This is because ES/GS interpretation is dependent on genetic ancestry. Variant interpretation upon which positive predictive values for ES/GS are measured has been established almost exclusively from individuals of European descent (Popejoy and Fullerton, 2016; Peterson et al., 2019). Given such underrepresentation of diverse ancestries, clinical interpretation of ES/GS results could be less reliable for newborns of non-European ancestry. Without adequate representation in datasets from individuals with diverse genetic ancestry, some newborns will benefit more from ES/GS than others. Clinical variant interpretation using resources such as ClinVar (Wain et al., 2018) and gnomAD (Gudmundsson et al., 2021) is therefore growing in importance, given they provide clinical assertions about genomic variants and associations with disease across genetically diverse populations. In general, problems of underrepresentation have prompted the development of new tools to monitor trends and identify gaps in genomic databases (Wang et al., 2022). Indeed, the global catalog of clinically actionable variants is expected to grow as reference data sets become larger, better curated and strive to be more representative of world populations.

### Re-Analysis and Obligations to Update Variant Interpretation

It is anticipated that routine re-analysis of “negative” screens might increase the diagnostic rate by 3%–5% per year and identify variants of concern in children who later present with clinical features suggestive of a genetic disease (Wenger et al., 2017; Costain et al., 2018). To capture these clinical benefits, NBS programs would need to systematically update screens and store ES/GS datasets in the health record to ensure results reflect up-to-date classification of genomic variants and take into account attendant costs and privacy risks. The treating physician may no longer be following the family and follow up with a new provider may be difficult and expensive. If a variant of uncertain significance were reclassified but not reported to the family based on clinical course, would NBS programs be subject to legal action if a child later manifests the disease (Clayton et al., 2021)? The expenditures and risks of storing all children’s genomic data long-term to enable such systematic re-analysis may also exceed those of re-sequencing only those children for whom it is clinically indicated in the future (Veenstra et al., 2021).

### Stigma, Psychological Impacts and Medicalization

Recent studies investigating the psychosocial impacts of expanding ES/GS in the newborn context have yielded different results. In a randomized trial of NBS with and without ES, researchers found both clinicians and parents valued information gleaned from standard of care NBS more than from exome sequencing but for different reasons (Pereira et al., 2019). Parents expressed knowing in advance how to prepare for a child with special needs was a benefit to sequencing, but worried about the psychosocial distresses brought on by variants of unknown significance and potential for discrimination among other things (Pereira et al., 2019). The potential for social stigma and medicalization of children with a molecular diagnosis who are pre-symptomatic (or destined never to exhibit the disease because it is non-penetrant) is also a concern. This scenario would be particularly concerning if enhanced surveillance or prophylactic treatments impinge on the child’s quality of life or expose them to interventions with adverse effects.

### Genomic Data Privacy and Protection

Key policy questions persist with respect to what rights and protections should apply to genomic and related health data collected at birth when newborns reach adulthood. The moral justification for mandatory NBS rests on the premise that finding the asymptomatic, at risk child is within the child’s best interests (United Nations Convention on the Rights of the Child, 1989). Child welfare considerations and the “the opportunity to intervene and dramatically alter a child’s life course and expectancy has been regarded as sufficient to preempt any claims of parental autonomy” (Goldenberg and Sharp, 2012). It is unlikely, however, that the huge volumes of data generated from ES/GS followed by untargeted whole exome/genome analysis will meet the criteria needed to justify overruling parental decision-making authority.

Yet samples taken from dried blood spots collected and stored using Guthrie cards are rich data sources needed to advance population health research. While most samples are de-identified or pseudonymized according to applicable laws/regulations when used for research, the generation of ES/GS data as part of NBS introduces novel ethical, legal and social challenges for data protection, agency and consent for the future adult (Khoury et al., 2003; Lewis, 2014). Genomic data are highly identifying and may implicate not only the individual tested but also their biological relatives. Concerns regarding loss of privacy and misuse of genomic data have emerged as key themes in the empirical literature on expansion of sequencing in NBS, and were found to be especially acute among participants of color (Joseph et al., 2016; Tsosie et al., 2021). It is unclear if the benefits of storing children’s genomic data in a centralized research data repository outweighs the privacy and security risks, particularly if children are not given the opportunity to consent themselves.

Re-consenting minors when they become adults to the continued use of their data collected at birth is supported in theory but logistically challenging to implement in practice (Knoppers et al., 2016; Rothwell et al., 2017; Nordfalk and Ekstrøm, 2019). Legislation passed in the United States in 2014, for example, requires that researchers seek broad consent for the use of the child’s dried blood spots for research beyond NBS (Newborn Screening Saves Lives Reauthorization Act, 2014). However this law preceded revisions to the United States Common Rule which now exempts research using de-identified data, thus removing a layer of specific consent (Lewis and Goldenberg, 2015; Rothwell et al., 2017). Empirical studies involving parents of both healthy and affected newborns suggest NBS programs should err on the side of greater transparency in terms of when, how and for what purposes their child’s samples and data will be used (Downie et al., 2021). Policy makers would need to determine whether, or how permissions for future use of ES/GS data for research will be incorporated into screening, and it remains unknown what effect this will have on public willingness to sustain state sponsored NBS programs that adopt ES/GS.

### ES/GS and the Wilson and Jungner Criteria

Disagreement regarding which disorders are screened for has largely (though not entirely) been avoided in some jurisdictions through standardization (Advisory Committee on Heritable Disorders in Newborns and Children, 2018) and concerted efforts are ongoing to harmonize screening lists internationally (Vittozzi et al., 2010; Franková et al., 2021). Wilson and Jungner anticipated such discrepancies and in 1968, developed criteria that outlined practical principles for screening services (Box 1) (Wilson and Jungner, 1968). While there have been recent calls to update the criteria to better align with technological advances in testing methods (King et al., 2021) and apply more nuanced decision analysis approaches (Prosser et al., 2012), the Wilson and Jungner criteria remain the generally accepted guidelines.

The threat to NBS participation should be a top concern if conditions are added to mandatory screening that challenge the Wilson-Jungner criteria or do not reflect how healthcare is accessed or paid for in a particular jurisdiction. Universal ES/GS with untargeted analysis in the NBS context poses several direct challenges to these criteria.

**BOX 1 | Proposed guide to screening for disease (Wilson-Jungner, 1968)**

1) The condition sought should be an important health problem.

2) There should be an accepted treatment for patients with recognized disease.

3) Facilities for diagnosis and treatment should be available.

4) There should be a recognizable latent or early symptomatic stage.

5) There should be a suitable test or examination.

6) The test should be acceptable to the population.

7) The natural history of the condition, including development from latent to declared disease, should be adequately understood.

8) There should be an agreed policy on whom to treat as patients.

9) The cost of case-finding (including diagnosis and treatment of patients diagnosed) should be economically balanced in relation to possible expenditure on medical care as a whole.

10) Case-finding should be a continuing process and not a “once and for all” project.

First, while there are many accepted treatments for conditions commonly screened for, most rare genetic diseases that are detectable by ES/GS do not have proven therapies.

Second, establishing a clinical diagnosis in an asymptomatic infant with a “molecular diagnosis” of a rare variant is resource-intensive, requiring specialized clinical assessment and variant interpretation, additional testing, and counseling services (Appelbaum et al., 2020). Newborn screening by any method should be accessible to every infant (Friedman et al., 2017; de Wert et al., 2021). To meet this universality target, healthcare centers must be equipped with appropriate sequencing infrastructure. Both human and material resources will therefore be needed in addition to those already allocated for existing NBS programs. At present, ES is available as a diagnostic tool primarily from certain clinical laboratories and through direct-to-consumer genetic testing services. A comparison of community report cards published by the National Organization for Rare Disorders (National Organization for Rare Disorders Newborn screening State report card, 2021) demonstrates that many NBS programs already face various resource limitations and vast differences exist in screening availability by U.S. states (Roman et al., 2020).

Disparities in NBS access and quality could be seen to violate the *parens patriae* doctrine which upholds that it is the duty of the State and its courts to protect the interests of persons in situations of vulnerability, for example children. NBS programs organized by the State are an extension of this duty (Knoppers, 1992), and the reasons many jurisdictions adopt an implied consent to NBS.

GS/ES-based NBS may well be different; if explicit consent is required, extant research suggests families are more likely to refuse consent, thus inadvertently denying their child the benefits of current NBS(Bombard et al., 2014; Joseph et al., 2016; Friedman et al., 2017; Genetti et al., 2019).

Moreover, the right of everyone to benefit from science and its applications is protected under Article 27 of the United Nations Declaration of Human Rights. While not a legally binding agreement, 193 countries have ratified at least one of the nine core international treaties which codify the Declaration’s commitments to basic rights and freedoms. Article 24 of the Convention on the Rights of the Child further obligates signatories to implement interventions that reduce infant and child mortality, to provide effective health care, and to combat childhood disease, among other legally binding responsibilities. Taken together, international conventions have been powerful tools for motivating the development and sustainability of public health programs (Reinbold, 2019) including NBS. Applying a human rights frame to the current debate favors expanding access to established NBS methods that have shown to be clinically effective, and which enable more children to directly benefit from proven methods. Ensuring universal access to high quality NBS irrespective of birthplace, gender and income, however, continues to be a global challenge (Krotoski et al., 2009; Borrajo, 2021).

Third, most genetic conditions diagnosed through ES/GS in early childhood have unknown natural histories or are unrecognizable during early childhood because the diseases are so rare and have only been described in a small number of patients.

Fourth, ES/GS is widely misunderstood among patients and clinicians alike, challenging overall public acceptance as a testing method. Issues of particular concern include data privacy, family decision-making when faced with an uncertain result and possible insurance discrimination (Pereira et al., 2019; Wojcik et al., 2021).

Fifth, recent analyses of global NBS coverage indicate that cost remains a barrier to even standard NBS access in low- and middle-income countries (Therrell et al., 2015, 2020; Howson et al., 2018; Therrell and Padilla, 2018). Since ES/GS cannot replace all current NBS by other methods, sequencing computing and storage costs for genomic data would be needed in addition to current laboratory costs to mitigate real privacy and security risks. Studies further show that clinical demand for medical geneticists and genetic counsellors far exceeds available services (O’Daniel, 2010; Boothe et al., 2021). Ultimately, however, NBS alone cannot reasonably be expected to universally improve health outcomes without addressing systemic health disparities, underlying social determinants of health (Melzer, 2022) and barriers to healthcare access (Goldstein et al., 2020) experienced predominantly by marginalized racial/ethnic groups (Sohn and Timmermans, 2019).

## Conclusion

Owing to the public health importance of universal access to NBS, applying ES/GS as screening tools in the newborn context is unsubstantiated as yet clinically and pragmatically. Ongoing translational research and technological advances will emerge in the coming years which are sure to improve our understanding of the opportunities and limitations of ES/GS in detecting and preventing early disease. Considering this evolving evidence, policy makers ought to be persuaded by a burden a proof that clearly demonstrates superior public health benefits of ES/GS beyond those achievable through traditional NBS methods. Attempts to concentrate efforts only on justifying the minimalness of any anticipated harms associated with ES/GS in NBS risks sidelining the real ethical, legal and social issues which have thus far tempered the promises of precision medicine in general.

Our position thus exposes a central tension in the debate between providing universal access to traditional NBS and respecting parents’ decision-making about much more extensive screening that they may perceive to be in the child’s best interests but that many adults may not opt for themselves. All screening programs expose individuals to potential harms that must be balanced against the benefits anticipated. This is not unique to genome-wide sequencing-based screening programs and is true even if only a selected “slice” of genes represented in the exome data were analyzed. The reality that some infants will screen positive and never experience symptoms does not justify excluding possible ES/GS for NBS. Rather the balance of benefits and harms must be quantified and considered in any policy decision regarding screening programs to ensure aggregate benefits outweigh foreseeable aggregate harms. Indeed, NBS programs must expand to provide all newborns access to screening that is of proven value, meet established criteria for proportionality (e.g., Wilson-Jungner) and shown to yield greater and more equitably distributed public health gains.

## Data Availability

The original contributions presented in the study are included in the article/Supplementary Material, further inquiries can be directed to the corresponding author.
